# Arthroscopic Repair of a "U" Shaped Rotator Cuff Tear: Modified Margin Convergence with a Single Triple-loaded Suture Anchor

**DOI:** 10.7759/cureus.6690

**Published:** 2020-01-17

**Authors:** Siddharth Jain, Sitender Garg, Ravi Mittal, Vijay Kumar Digge, Ashish Shukla, Ganesh V

**Affiliations:** 1 Orthopedics, All India Institute of Medical Sciences, New Delhi, IND; 2 Orthopedics, Indian Army, New Delhi, IND

**Keywords:** rotator cuff tear, marginal convergence, triple loaded suture anchor

## Abstract

Introduction

Repair of a “U” shaped rotator cuff tear tends to create extreme tensile forces at the central part of the rotator cuff margin, causing tensile overload and may result in subsequent failure. We describe our technique of repairing the “U” shaped tear in which margin convergence is done using Ethibond (Ethicon Inc., New Jersey) and a single triple-loaded suture anchor. It results in the reduction of the strain and also allows the repair of seemingly irreparable tears.

Patients and method

We included 10 patients having a “U” shaped degenerative rotator cuff tear. All patients were assessed preoperatively. The University of California at Los Angeles Shoulder score (UCLA shoulder score) recorded preoperatively and at final follow-up was used to assess functional outcome.

Result

Out of 10 patients, six were males and four were females. The mean age was 60 years (range 50-70 years). The average follow-up was for 31 months (range 24 - 48 months). The UCLA score increased from an average of 9 preoperatively (range 8 - 12) to an average of 29.6 (range 27 - 31) at the terminal follow-up. The UCLA increased in the postoperative period and was statistically significant (unpaired t-test; p < 0.0001). All patients had good/excellent outcomes (UCLA score > 27). Abduction increased from average 27 degree preoperatively (range 20 degree - 35 degree) to an average 131 degree (range 125 degree - 140 degree) at final follow-up (unpaired t-test; p < 0.0001).

Conclusion

Our technique of modified margin convergence achieves low tension repair and anatomical footprint reconstruction with good clinical outcomes using a single triple-loaded anchor.

## Introduction

Tears in the rotator cuff are one of the commonest causes of shoulder pain, particularly in elderly patients [1]. The rotator cuff tear size generally increases over time. Many symptomatic patients may require surgery once the trial of conservative treatment fails. The cuff tears have been classified according to the geometric tear pattern as a crescent, longitudinal “U” shaped, or “L” shaped, massively contracted, and irreparable tear associated with rotator cuff arthropathy [1]. Arthroscopic repair of the rotator cuff has been a well-known surgical procedure to reconstruct the cuff integrity. Still, the treatment of patients having large or massive cuff tears is always a challenging procedure. A “U” shaped tear has a greater medial extent with the apex medial to the glenoid margin when compared to a crescent-shaped tear. Identifying the tear pattern is crucial because the medial to lateral mobilization of a “U” shaped tear tends to create extreme tensile forces at the central part of the repaired cuff margin, causing tensile overload and may result in subsequent failure [2]. The technique of “margin convergence” was first introduced by Burkhart. This technique includes side-to-side approximation using suture from medial to lateral of the anterior and posterior limb of the tear, causing the free margins of the tear to converge toward the bone bed on the greater tuberosity [3]. It decreases the strain across the tear, which allows the successful repair of a large and massive “U” shaped tear. A side-to-side approximation of the medial two-thirds of a “U” shaped tear reduces the amount of strain at the tear margin by a factor of six, thus securely fixing the cuff and protecting the repair site from failure [4].

We describe our technique of repairing the “U” shaped tear in which only margin convergence is done using a single triple-loaded suture anchor.

## Materials and methods

We included 10 patients having a “U”-shaped degenerative rotator cuff tear in this retrospective study. All the patients underwent surgery between December 2015 and December 2017. We excluded the patient with a crescent-shaped tear, “L” shaped, or reverse “L” shaped tear. We also excluded patients with diagnosed inflammation. All patients had undergone a preoperative assessment. The clinical presentation in all cases was pain and restriction of active movements of the shoulder joint with the preservation of a passive range of movements (pseudo paralysis) of varying duration. The University of California at Los Angeles Shoulder (UCLA) shoulder score [5] was recorded preoperatively and at the final follow-up. It was used to assess functional outcome. All operative procedures were performed by the same senior surgeon.

Operative technique

**Figure 1:**
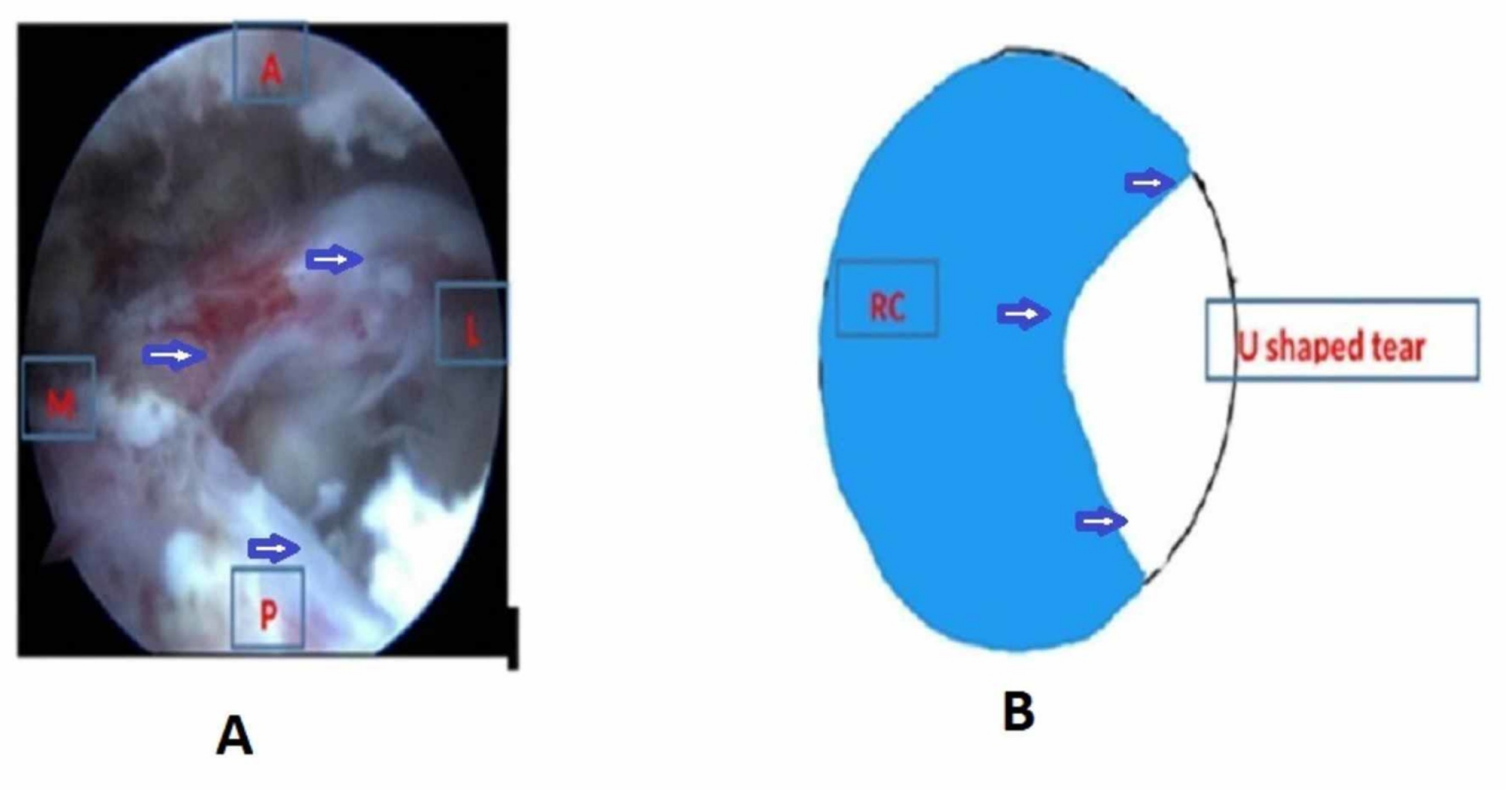
U-shaped tear in the right shoulder seen from the posterolateral portal A - Arthroscopic picture; B - Animated picture; Blue arrow - Showing rotator cuff tear margin; RC - Rotator cuff; M - Medial; L - Lateral; A - Anterior; P - Posterior

**Figure 2:**
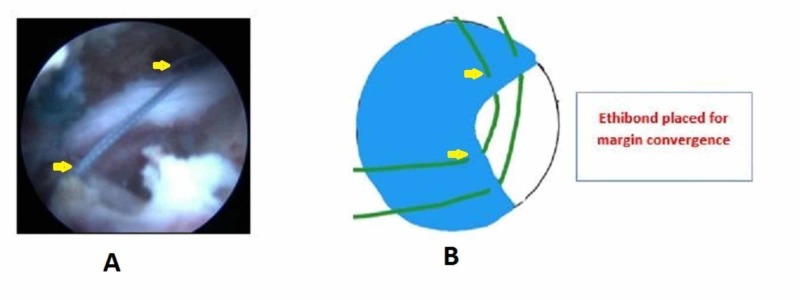
Starting from medial to lateral, two margin convergence sutures passed in the anterior and posterior limbs of the tear A - Arthroscopic picture; B - Animated picture; Yellow arrow showing Ethibond sutures through the anterior and posterior margins of the cuff tear for margin convergence

**Figure 3:**
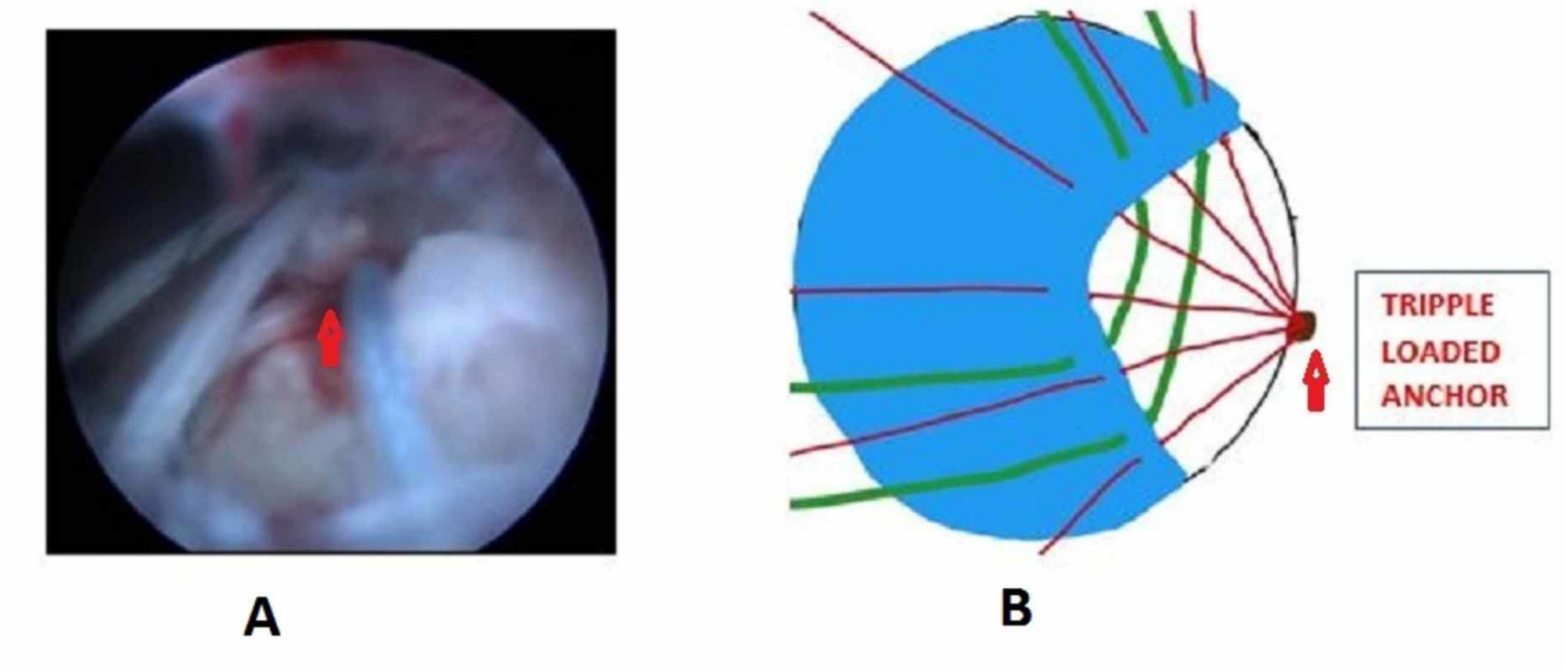
A triple-loaded suture anchor inserted in the footprint and sutures passed in the anterior and posterior limbs of the tear A - Arthroscopic picture; B - Animated picture; Red arrow showing a triple-loaded suture anchor

**Figure 4:**
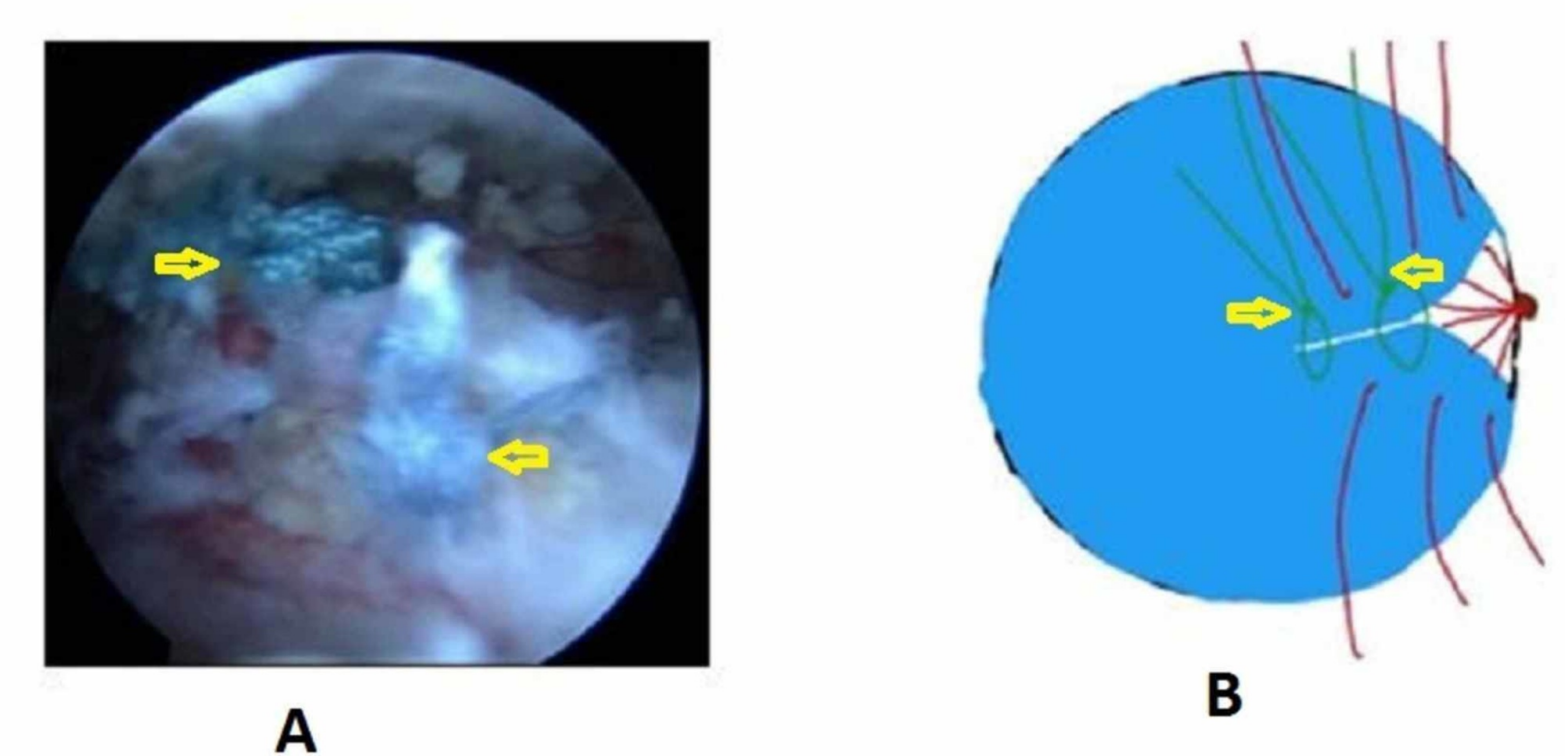
After knot tying of the two medial free sutures A - Arthroscopic picture; B - Animated picture; Yellow arrow showing margin convergence after tying of Ethibond sutures Ethibond: Ethicon Inc., Somerville, New Jersey

**Figure 5:**
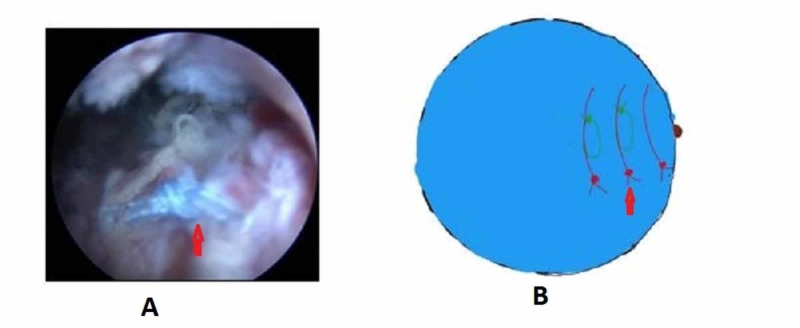
Knots tied in all three sutures from the anchor; complete repair of the tear with full coverage of the footprint A - Arthroscopic picture; B - Animated picture; Red arrow showing an approximation of the rotator cuff tear margin to the footprint after tying of the triple-loaded anchor sutures

We positioned the patient in lateral decubitus with the arm abducted to 30 degrees and forward flexion to 30 degrees after general anesthesia. Posterior, anterolateral, lateral, and posterolateral portals were used. The arthroscope was inserted into the posterior portal and a diagnostic round was performed. Additional lateral, anterolateral, and posterior-lateral portals were made to debride the subacromial bursa. The site and extent of the rotator cuff were visualized after bursectomy and acromioplasty. The pattern of all tears was U shaped (Figure 1). The apex of the tear could not be brought to the footprint on the humerus by lateral traction. The anterior and posterior limbs could be brought near each other. Through the lateral portal, Expressew II (flexible suture passer; DePuy Mitek Inc., Raynham, MA) was passed to put Ethibond No. 5 (Ethicon) through the anterior and posterior limbs of the tear. Two such sutures were passed as shown in Figure 2. Then, a corkscrew FTIII suture anchor (Arthrex, Naples, Florida), 5.5 mm X 16 mm with three number 2 fiber wire was placed percutaneously midway between the medial and lateral margins of the footprint. The two limbs of each suture were passed into the anterior and posterior leaflets of the cuff tear (Figure 3). The sutures were then sequentially tied from the medial to lateral side (Figure 4). As the sutures were tied, the margins of the cuff tear converged, the anteroposterior gap in the tear decreased, and the free margin of the cuff moved towards the footprint. After tying all the sutures, it was found that there was no gap in the leaves of the cuff and the cuff had covered almost the whole of the footprint (Figure 5).

## Results

There were six males and four females. The average age was 60 years (range 50-70 years). The average follow-up was 31 months (range 24 - 48 months). The average tear size was 2.7 cm (2.4-3 cm). The UCLA score increased from an average of 9 preoperatively (range 8 - 12) to an average of 29.6 (range 27 - 31) at the final follow-up (Table 1). The UCLA score was found to be improved post-operatively and was statistically significant (p < 0.0001). All patients had good/excellent outcomes (UCLA score > 27). Abduction increased from an average of 27 degree preoperatively (range 20 degree - 35 degree) to an average 131 degree (range 125 degree - 140 degree) at final follow-up (p < 0.0001). The average visual analog score (VAS) decreased from an average of 4.5 preoperatively (range 3 - 6) to an average of 0.3 (range 0 - 1) at the final follow-up (p < 0.0001).

**Table 1:** Patient demographics, tear size, UCLA score, abduction, VAS at final follow-up UCLA - University of California at Los Angeles shoulder score; VAS - Visual analog score; Preop - Preoperative; Postop - Postoperative

Serial no.	Age	Sex	Tear size (mm)	UCLA score		Abduction (in degree)		VAS	
				PREOP	POSTOP (at final follow-up)	PREOP	POSTOP (at final follow-up)	PREOP	POSTOP (at final follow-up)
1	50	F	2.6	8	31	0-25	0-140	5	0
2	58	M	3.0	10	29	0-30	0-130	5	0
3	70	M	2.6	12	30	0-35	0-135	6	1
4	63	M	3.0	8	27	0-20	0-125	4	1
5	67	M	2.8	8	31	0-25	0-135	4	0
6	58	F	2.7	8	31	0-20	0-130	5	0
7	53	F	2.4	10	30	0-35	0-135	4	0
8	54	F	2.8	10	28	0-35	0-125	5	1
9	65	M	2.5	8	29	0-20	0-125	4	0
10	62	M	2.6	8	30	0-25	0-130	3	0

## Discussion

Successful repair of large and massive "U" shaped rotator cuff tears with a good functional outcome still remains challenging. With the recent advancement in technology and with emerging techniques, such tears can be repaired with good results. Newer techniques include margin convergence, shoelace convergence, massive cuff stitch, transosseous-equivalent, suture bridge, double-row suture anchor configuration, over-under lacing technique, and so on. Arthroscopic repair of a large “U” shaped tear by fixing tendon to the bone using suture anchors alone may result in high tension at the tendon-bone junction and can lead to a failure of repair. Before Burkhart described the technique of “margin convergence’ for the repair of “U” shaped tears, these were routinely treated with repair to the bone under tension or repairing them with the arm in undue abduction [6]. These repairs had reported a significantly high failure rate. The introduction of the technique of “margin convergence” has revolutionized the arthroscopic repair of large and massive cuff tears by reducing undue tension on the repair site.

The biomechanical principle upon which “margin convergence” works has been thoroughly described by Burkhart [3,6-7]. It has been shown that side-to-side approximation of the anterior and posterior margins of the tear using sutures significantly reduces strain on the free margin of the cuff, as it decreases the length of the tear (mediolateral length) as well as increases the healing surface area. This reduction in strain across the approximated margins allows the cuff to be repaired onto the bone successfully with suture anchors and protects the repair [6-7]. Burkhart et al. first published the clinical results of margin convergence in large and massive cuff tears in 2001 [7]. The difference between tears repaired directly to the bone and those treated with margin convergence (with or without suture anchors) was found to be insignificant. This was the first report that showed that irrespective of the size of the tear, good results can be achieved if large/massive tears were managed by margin convergence. Unlike the technique described by Burkhart et al, We did not convert a “U” shaped cuff tear into a crescent-shaped cuff tear and then fix the cuff to the bone using suture anchors; rather, we did margin convergence of the whole tear and cover the whole footprint.

Mazzocca et al., in their biomechanical evaluation, have shown that there was a significant reduction in strain and gap size in rotator cuff after performing margin convergence for a large size cuff tear [8].

Jones et al., in a study involving 60 large or massive tears, concluded that a combination of one or more margin convergence sutures and one or more suture anchors to repair the cuff was the best technique to repair such tears [9]. Most patients have achieved good and excellent results. They also opined that residual gaps following repair do not adversely affect outcomes as long as force couples are maintained. Shindle et al. used both single-row and double-row configurations with multiple anchors for marginal convergence and concluded that margin convergence is a useful technique for repair of U-shaped cuff tears which are difficult to mobilize [10].

A modified margin convergence technique was also described by Kim et al. in 15 massive cuff tears [11]. In their opinion, the conventional method of margin convergence may lead to incomplete footprint reconstruction. Their technique was similar to ours, except that they did a double-row repair with two double-loaded anchors. They believed that using suture anchor threads in a margin convergence fashion and a double-row repair with two anchors results in a more anatomical footprint reconstruction. The results of our technique show that a single triple-loaded anchor also achieves the same goal.

The over-under lacing technique for modified margin convergence has been introduced by Metais et al. [12]. They found good clinical results in patients having a “U” shaped massive cuff tear. In another study, Ryu et al. described a modified suture bridge technique, in which a horizontal mattress suture is passed at the lateral free end of the cuff margin to complete the cuff repair. Single-stitch marginal convergence repair along with footprint repairs can lead to increased muscle stiffness; this may result in increasing the tensile loading on the cuff repair site. By adding multiple marginal convergence sutures, these adverse effects can be avoided [13]. In our technique, we used multiple marginal convergence suture for the "U" shaped cuff tear to approximate anterior and posterior margins and found satisfactory clinical results.

Advantages of this technique include:

1. Low cost (one suture anchor used).

2. The triple loaded anchor is biomechanically superior to the double-loaded anchor (more bites per anchor through the rotator cuff).

3. The anchor helps achieve both margin convergence and footprint restoration.

The limitations of our study include the retrospective study design, low sample size, lack of postoperative ultrasonography (USG) or magnetic resonance imaging (MRI) to assess the rate of healing, and the exclusion of acute traumatic tears in younger patients. Although future long-term prospective studies comparing our technique with the classical technique described by Burkhart are needed to establish the reliability and safety of our technique, we believe that the preliminary results presented in this study are encouraging.

## Conclusions

Our technique of modified margin convergence achieves a low tension repair and anatomical footprint reconstruction with good clinical outcomes using a single triple-loaded anchor. Our technique for the repair of a “U” shaped cuff tear using marginal convergence is simple to learn and reproduce.
